# Preventing overloaded dissemination in healthcare applications using NonDelay tolerant dissemination technique

**DOI:** 10.1016/j.heliyon.2023.e18783

**Published:** 2023-07-28

**Authors:** Mohd Anjum, Sana Shahab, Taegkeun Whangbo, Shabir Ahmad

**Affiliations:** a Department of Computer Engineering, Aligarh Muslim University, Aligarh, 202002, India; b Department of Business Administration, College of Business Administration, Princess Nourah Bint Abdulrahman University, PO Box 84428, Riyadh, 11671, Saudi Arabia; c Department of Computer Engineering, College of IT Convergence, Gachon University, Seongnam, 13120, Republic of Korea

**Keywords:** Data dissemination, Healthcare applications, Non-delay tolerant, Wearable sensors

## Abstract

Wearable Sensors (WSs) are widely used in healthcare applications to monitor patient health. During the data transmission, dissemination requires additional time to transmit the details with minimum computation difficulties. The existing techniques consume high overloaded while transmitting data in healthcare applications. The research problem is overcome by applying the non-delay-tolerant dissemination technique (NDTDT) to prevent overloaded dissemination and augment immediate, swift message delivery. The dissemination techniques utilize the intelligent decision-making process to provide the accumulated details to the healthcare center. The proposed approach is reliable in mitigating the errors due to inconsistent and discrete sensing intervals between the WSs. The constraints due to delay and interrupted transmission losses are reduced by selecting appropriate slots for WS information handling. This technique aims at maximizing the delivery of accumulated WS information through non-submissive or underlay dissemination. The method is designed to reduce dissemination delay and maximize successful message delivery. Two variations, sensors and data flows, validate the proposed NDTDT system's performance. The model increases the delivery rate by 0.91% and 0.932%, the dissemination probability by 0.964% and 0.98%, and the final metrics involved are an average delay of 12.78 ms and 11.67 ms.

## 1 Introduction

In recent years, wearable sensors (WS) have been prevalent in healthcare and have revolutionized healthcare services, providing real-time, personalized data to patients and healthcare professionals. This real-time data has significantly improved the healthcare system efficiency and patient outcomes and reduced healthcare costs [1]. Therefore, they have augmented a sophisticated contribution to healthcare and medical diagnosis [2]. WS are tiny electronic communication on-chip devices worn as gadgets by the end-users to monitor various physiological or environmental parameters [3]. These sensors are placed over the skin surface or attached to the wearable clothing of the end-users [4]. The purpose of these sensors is specific such as reliable monitoring of physiological changes in the human body [5,6]. These sensors sense parameters such as oxygen saturation, heart rate, blood pressure, physical activity level, etc., and transmit the captured data wirelessly for clinical analysis [7]. As the design of the sensors facilitates radio communication and sensing modules, transmitting the sensed information to the destination is automated and controlled through a handheld mobile device [8,9].

Sensors such as electrocardiography, thermistor, electromyography, etc., are fabricated as wearable watches, belts, rings, etc. [9]. The sensors have many medical applications, such as the detection of biological activities [10], diagnosis of diseases [11], and bio-components detection [12]. The functions of the devices are battery-operated, with which they perform sensing and dissemination instances. WS communicates in random intervals as the sensing of physiological changes depends on the end-user activities. The heart rate monitoring sensor should transmit the data during exercise or any uneven changes in heart rate rather than transmitting data continuously [13]. Sensed parameters are classified based on priority depending on the physiological changes observed. The information disseminated from the sensors is acquired by the handheld mobile device that relays the same to a faraway clinical center. Therefore, wearable devices are integrated with sophisticated interoperable technologies, allowing them to sense and transmit data seamlessly and efficiently [14].

Communication support of the WS is interoperable, allowing them to reach different connected devices for information delivery [15]. This interoperability enables the WS to communicate with various devices, such as handheld devices, healthcare devices, and systems. Sensor data dissemination is crucial for improving analysis reliability in healthcare applications [16]. Loss or interruption in data dissemination produces improper analysis and partial diagnosis in healthcare applications. Now, ensuring that the disseminated data must be accurate, timely, and complete is mandatory. Therefore, the dissemination model designed for WS technologies is interoperable and adaptable to support multiple communications simultaneously [17]. The wearable sensing technology supports the existing communication standards, such as ZigBee, Wi-Fi, 802.11, Bluetooth, etc., for a wide range of sensed data dissemination [18]. This technology also coexists with the sophisticated cloud and other information technology paradigms for easy access and information sharing [19,20]. The Internet of Things (IoT) is a powerful healthcare application platform transforming healthcare services [21]. IoT enables the interconnection of physical devices, sensors, and systems, allowing them to communicate and share data [22]. IoT is used to improve the efficiency of healthcare by developing the transmission, storage, and processing of the output data of wearable sensors in real-time, enabling healthcare providers to make informed decisions and provide better patient care [23]. Furthermore, artificial intelligence techniques such as machine and deep learning are prevalent and efficient for medical applications [24]. These techniques are integrated with WS to enable classification and pattern identification approaches that improve the accuracy and efficiency of medical diagnosis and treatment [25].

Now, an effective technique is needed to manage communication reliability. This paper proposes the Non-Delay Tolerant Dissemination Technique (NDTDT) as an effective method for managing communication reliability. The NDTDT approach effectively addresses issues related to slot synchronization and congestion flow in data transmission from wearable sensors. The major objectives of this paper are stated as follows:

• To prevent overloaded dissemination, augment immediate and swift message delivery using NDTDT.
• To reduce the delay and interrupted transmission losses by selecting appropriate slots for WS information handling.
• To reduce dissemination delay and increase the delivery of accumulated WS information.
• To resolve slot synchronization and congestion flow problems in data transmission.

The manuscript is arranged as follows: Section 2 discusses the related work in healthcare application communication-related parameters and a summary table with various metrics. Section 3 implements the NDTDT technique. Section 4 discusses the results and discussion with comparative analysis. Section 5 concludes the work with limitations and future scope.

## 2 Related work

In the last decade, a significant shift has been seen toward developing more connected and data-driven ecosystems. The backbone of these ecosystems is advancement in wireless communication technologies, reduced electrical components power usage, and the emergence of autonomous sensing systems. These autonomous systems can generate, transmit, and receive important information in various fields, such as healthcare, smart homes, and transportation. Therefore, many smart wearable devices have been developed to monitor physiological parameters. Manas et al. [26] developed a wearable health monitoring and messaging system with an Android interface based on IoT. This system was used to detect medical distress in a person, and a message with the person's location was automatically sent to the caregiver or doctor to get the right attention. Compared with a traditional photoplethysmography circuit, it was designed to prevent discrete components. I and Cai [27] introduced a delay-constrained scheduling method (DCSM) for medical data packet transmission in an IoT-based healthcare network. The implemented network was based on wireless body area network (WBAN) communication which comprised a gateway, medical centers, and base stations. The DSCM model primarily focused on beyond-WBAN communications. When the delay-constrained was beyond WBAN, the transmission request was sent to the intelligent network controller et al. [28] utilized WBAN to monitor patient health. The author offered a medical network to observe the sensor nodes' vital signs. The nodes aim to analyze the respiration rate, heart rate and body temperature using the photoplethysmography sensors.

In [29], authors proposed constrained Markov decision process-based intelligent transmission for remote health monitoring. The introduced scheduling algorithm reduced control problems between the wireless sensor links to optimize energy efficiency. During the analysis Markov decision process examined the decision while efficiently transmitting information from source to destination. Yi and Cai [30] proposed an E-health network based on delay-dependent aware transmission (DDPT). The algorithm aggregated the medical packets based on their criticality and experienced delays to transfer via the WBAN. Information priority was examined during the analysis to transmit the details with the minimum computational requirement. This algorithm applied dynamic priority discipline with a virtual delay-dependent for queuing the data packets. Zhang et al. [31] addressed the WBAN cost optimization problem while transmitting data over the network. The transmission occurs through limited energy capacity, and the monitoring devices and base stations control the data exchange costs. Optimizing a distributed decision-making algorithm for transmission costs was designed for artificial intelligence. In Ref. [32], authors presented a dual sink approach using clustering in the body area network for routing the data packets. It was more reliable, stable and energy-efficient than its counterparts. This protocol enhanced the network lifetime by presenting the idea of clustering using two sink nodes.

Zhang et al. [33] determined that wearable devices reduced energy usage for data transmission in time constraints by combining data compression and wireless transmission speed control. The approach was tested in both an offline and a stochastic setting, and an efficient approximation algorithm and a stochastic algorithm were presented to minimize the total expected energy usage. Shu et al. [34] introduced a mobile edge-aided data transmission (MEC-DT) model for health care. The proposed model used the mobile edge servers to disseminate data packet segments and then exchange them among IoT nodes to reassemble the whole packet. The installation mechanism and protocol for data propagation ensured the integrity of the transmitted data objects to avoid transfer conflicts. An adaptive protocol then achieved efficient data dissemination for heterogeneous IoT networks. An and Chung et al. [35] proposed a single-LED multiple-channel transmission system using visible light communication for electromagnetic wave-free indoor healthcare monitoring. This system applied sparse code multiple access to transmit the medical data in real-time with high bandwidth. The multi-user data was spread to the temporal domain using a non-orthogonal codebook and a single LED illuminated by a photodiode. Bhatia and Patro et al. designed a poll-based media access control protocol for WBAN [36]. It was applied to meet the diversified function requirements of various WBAN applications. The author introduced a few concepts in the polling-based channel access mechanism to make an energy-efficient and quality-of-service-aware media access control protocol.

Arul et al. [37] suggested a blockchain-based multi-modal secure data dissemination framework to ensure patients retain control of their personal health information. The proposed framework offered the highest level of security and privacy, significantly benefiting healthcare data management in IoMT devices. A healthcare application network, secured via blockchain technology, has been developed, allowing patients' medical records to generate important signals for verified healthcare experts. Federated learning [38] could solve the challenges in healthcare with a centralized aggregate server disseminating a global learning model. The local participant maintains authority over patient data, preventing unauthorized users. In Ref. [38], the authors presented a systematic literature review about the interaction of blockchain technology with federated learning in the Healthcare 5.0 system. This research aimed to build a blockchain-based intrusion detection system-enabled health monitoring system that would allow physicians to stay updated on the patients via sensors and take preventative actions in real time using disease forecasting. In Ref. [39], the author developed a method for transferring knowledge based on data, using machine learning algorithms to analyze data stored on the mHealth server. Initially, the authors performed some statistical analyses on the data using techniques such as logistic regression, neural networks, k-nearest neighbors, and support vector machines. Then, they employed a hidden Markov model to reveal previously unknown patterns in the data. Finally, the authors established fuzzy rules for sharing information with consumers. After the above analyses of various related studies, a comparison of these studies based on different parameters is demonstrated in Table 1.

**Table 1:** Comparison of related works based on various metrics.

Year	Ref.	Methodologies	Problem Definition	Dataset used	Results	Limitations
2019	[26]	The wearable digital filter device	To avoid the use of discrete components	Online healthcare	Improved accuracy and minimal time consumption	mobile must be internet enabled
2019	[27]	delay-constrained scheduling method (DCSM)	To characterize the dynamic nature of wireless transmission	Remote healthcare database	Network revenue, packet transmission ratio	Hardware constraints
2018	[28]	Small low, power wearable device	To maintain long-term usage	Daily basis IoT based monitoring	Respiration and Heart rate	Lack of power management strategies
2020	[29]	Markov decision, Lagrange multiplier approach	To optimize the energy efficiency of the WBAN link	E-healthcare service	Throughput, better power consumption	
2019	[30],	delay-dependent aware transmission (DDPT)	Focused on transmission schedule and starving time	E-healthcare networks	Reduced waiting delay cost,	Lack of information on concurrent transmission
2019	[31],	Distributed optimization model	Power cost optimization challenges	Medical Surveillance network	Energy cost reduction	–
2017	[32],	dual sink approach using clustering	To cover the line of sight and non-line of sight problems	–	Network throughput, lifetime, delay, and stability	Lack of resource efficiency parameter
2018	[33],	efficient approximation algorithm and a stochastic algorithm	Focused on energy usage	–	Energy consumption	Scalability issues
2019	[34],	mobile edge-aided data transmission (MEC-DT)	To avoid energy wastage	Healthcare service	Improved energy efficiency	Network latency impacts
2018	[35],	the single-LED multiple-channel transmission system	To monitor multiple medical data	Indoor Healthcare monitoring	Higher bandwidth efficiency	Simple structure for practical implementation
2018	[36],	poll-based media access control protocol	Focused on network lifetime	–	Minimal delay and high reliability	Resource utilization challenges
2021	[37],	blockchain-based multi-modal secure data dissemination framework	Focused on secure access to patient information	Healthcare application	High accuracy, prediction, less delay, latency, and response time	processing power limitations
2022	[38],	Federated learning	To improve the clinical learning process	Healthcare network	accuracy	Computational complexity
2022	[39],	logistic regression, neural networks, k-nearest neighbors, support vector machines, hidden Markov model	Focused on real-time feedback	mHealth service	Improved accuracy	Integration challenges

According to the different research results, an effective technique should be incorporated to improve communication reliability. The Non-Delay Tolerant Dissemination Technique (NDTDT) is introduced in this work to achieve the goal mentioned earlier. The NDTDT method can resolve slot synchronization and congestion flow problems in wearable sensor data transmission. The suggested NDTDT is designed to optimize the transmission of accumulated WS data and messages while minimizing the time to disseminate the information.

## 3 Non-Delay Tolerant Dissemination Technique (NDTDT)

**Fig. 1:**
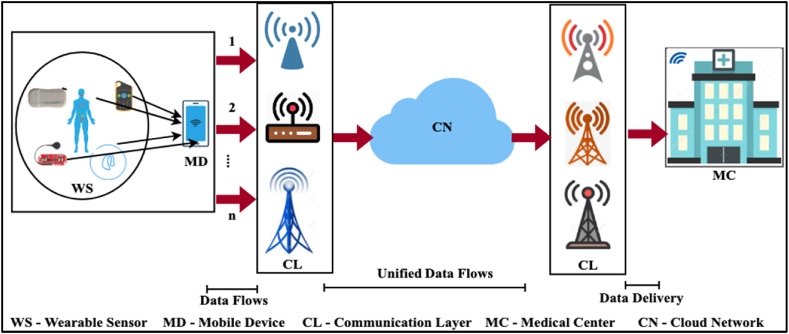
Ndtdt in a Typical WS-MC architecture.

The NDTDT design aims to identify reliable communication devices for transmitting medical sensor data. Despite congestion flows and transmitting slot synchronization challenges, wearable sensor data must be delivered for processing in time. The proposed transmission technique is designed considering the significance of wearable sensor data and its response to the end-user. Unlike conventional transmission techniques, decision-making is performed in end-to-end decision-making, and transmission delay and congestion factors are jointly assessed. Besides, the priority of the wearable sensor message determines the significance of handling medical data. In Fig. 1, the dissemination technique endorsed architecture is portrayed.

Fig. 1 presents a communication model, integrating a communication layer (CL) for data collection and dissemination. The mobile device (MD) aggregates the data flows from the wearable sensors (WSs). The CL present at both the MD and Medical Center (MC) ends is connected through a common platform such as a cloud network (CN). Multiple data flows (1 to n) between MD and CL are unified as a time-based workflow stream in CN. MC receives a signal stream of data in different time intervals. The rate of change $\left(\frac{d_{d}}{d_{a}}\right)$ must be increased regardless of the time intervals. The data delivery and data aggregated rate from the wearable medical sensors are represented by the variables $d_{d}$ and $d_{a}$. Delay (transmission) and delivery modeling based on the decision-making process are the core constituents of NDTDT. Communication systems like healthcare scenarios can deliver data faster, improving responsiveness and timeliness by lowering transmission delay. Communication systems can lessen the consequences of traffic overload by taking congestion into account and controlling it, ensuring more dependable communication channels and smoother data transmission. Through delays, a higher risk of data loss, and a reduction in overall service quality, congestion can substantially negatively influence the reliability of communications. By efficiently handling these aspects, communication systems can improve data transfer reliability and satisfy various applications' unique requirements. Delay and delivery modeling through linear decision-making is explained in the following sections.

### 3.1 Delay modeling

The priority of the active sensors is calculated using the parameter $d_{a}$ before certifying the transmission delay of medical data. For example, an ECG sensor is prioritized highly compared to a temperature monitoring sensor given by the $d_{a}$. The value of k represents the medical data transmitted in a time interval $t_{t}$.The medical data is generated from the wearable sensors $S_{n}$ in which the data flow of k is calculated using $\left(f\times d_{a}\right)$ denotes the aggregated information flow at different times. During the data transmission process, the delay time $\left(t_{del}\right)$ is estimated using Equation (1).

(1) $$t_{del}=\sum_{i=1}^{k}\left[\frac{{\left(t_{t}-t_{d}\right)}_{i}\times p_{i}}{{\left(f\times d_{a}\right)}_{i}}\right]$$

From Equation (1), the delay time can be calculated because the signal flow rate of medical data is represented as $\left(f\times d_{a}\right)$ for medical data k given in a time interval for data transmit $t_{t}$ and time of data delivery $t_{d}$ based on the priority p of medical data. The difference between the time of transmission and delivery of data can be easily identified; hence, the delay time can be calculated using Equation (1). In Equation (1), f={1,2,…,n} denote the $d_{a}$ flows at different times $\left\{t_{1},t_{2},\ldots ,t_{n}\right\}$. In each $t_{n}$, the $\left(f\times d_{a}\right)$ is the aggregation rate that is relayed through MD the transmission and delivery times of $d_{a},p$ is the priority of the medical data given by $t_{t}$ where t represents the transmission of data in a time interval t and $t_{d}$ represents the delivery of data in a time interval. k is the number of medical data transmitted in $t_{t}$. Here, p is estimated as $\frac{S_{n}-\frac{k}{2}}{\left(\frac{k}{2}+1\right)}\forall \frac{k}{2}<S_{n}<k\text{.}$ The above Equation is computed for k generated from wearable sensors $S_{n}$. Besides, the time is computed for a signal flow rate of $\left(f\times d_{a}\right)$ for k medical data. The objective here is framed as follows:

(2) $$\begin{array}{c} \sum_{i=1}^{n}\mathrm{argmin}\sum_{j=1}^{k}t_{{del}_{j}},n\in f\\ \frac{d_{d}}{d_{a}}\;is\;maximum\;and\\ \begin{array}{c} t_{del_{1}}=t_{del_{2}}\;or\\ t_{del_{1}}-t_{del_{2}}=minimum,minimum<t_{t} \end{array} \end{array}\}$$

Equation (2) emphasizes the maximization of $\frac{d_{d}}{d_{a}}$ and confines the time difference between $t_{{del}_{j}}$ within $t_{t}$. Here, n represents multiple data flows starting from counting one. $t_{del_{1}}$ and $t_{del_{2}}$ are two instances of j where it ranges from 1 to k. It helps to improve the flow of medical data under controlled delay. To improve the medical data, etiquette, and transmission rate at $t_{t}$ of $\left(f\times d_{a}\right)$, the states of the dissemination are defined. There are two states in dissemination, namely [halt (h) and transmit (t)]. The decision process follows the Markov model, where the transaction (T) of the states is estimated by analyzing the current and next state defined in Equation (3).

(3) $$T=\rho \left[h_{t_{t}+1}=t|h_{1}=h\right]+\rho \left[t_{t+1}=t|t_{t_{t}+1}=h_{t_{t}+1}\right]$$

In Equation (3), the probability of a halt to transmit state is defined for all the flows such that the objective is to achieve maximum t throughout $t_{del}$. Here, the next state value ($h_{t_{t}+1})$ is estimated as, $\left[\begin{array}{c} \begin{array}{ccc} h_{11} & \cdots & h_{1t_{t}} \end{array}\\ \vdots \\ \begin{array}{ccc} h_{f1} & \cdots & h_{ft_{t}} \end{array} \end{array}\right]\left[\begin{array}{c} \begin{array}{ccc} t_{11} & \cdots & t_{1t_{t}} \end{array}\\ \vdots \\ \begin{array}{ccc} t_{f1} & \cdots & t_{ft_{t}} \end{array} \end{array}\right]$. The state of t retains the action of $\left(f\times d_{a}\right)$ dissemination across all the available communication layer units. It helps to confine delay without frequent change in T, reducing the data loss rate. The process of determining the T for multiple instances, the profit of the previous transaction $\left(\tau_{T}\right)$ is estimated. If the profit is high, $\rho \left[h_{t_{t}+1}=t\right]$ is retained else, the state is transacted $t_{t_{t}+1}=h_{t_{t+1}}$. By considering the data loss rate in the previous transaction, the error value is calculated concerning all transactions that happened before from the initial state with minimum error from halt to transmit state and maximum count of successful transmit happened for each state and maximize the data delivery and data aggregated value from sensor $S_{n}$ states the aggregated data flows at different time intervals based on aggregated data. The profit is estimated as follows.

(4) $$\tau_{T}=\frac{1}{T}e\left[\sum_{i=1}^{T}\frac{Tdel{\left(h_{t_{t}+1}=t\right)}_{i}-\rho \left[h_{t_{t}+1}=t\right]}{\left(S_{n}\times f_{i}\times d_{ai}\right)}\right]$$

**Fig. 2:**
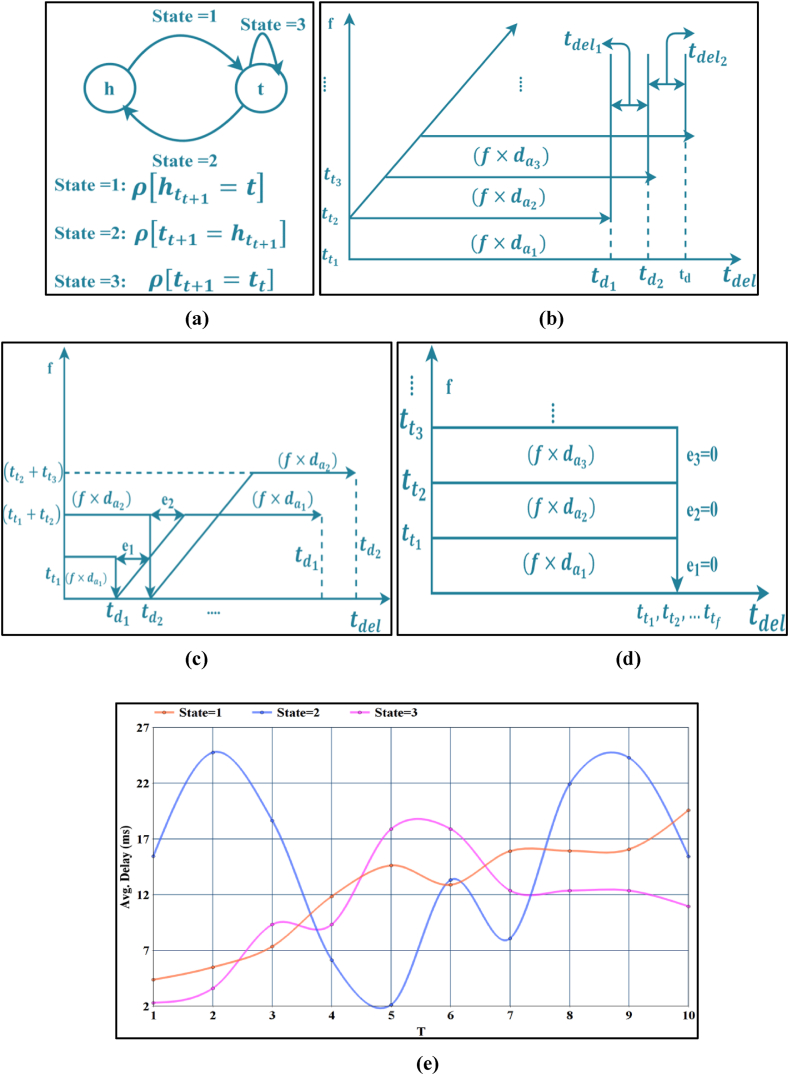
State transaction representation (a) State Transactions; (b) State = 1 transaction; (c) State = 2 transactions, and (d) State = 3 transactions. (e): Avg. Delay for varying T.

In Equation (4), e denotes the error in $t_{t}$ (i.e.) the $\left[\frac{t_{del_{1}}-t_{del_{2}}}{count\left(t_{del_{1}}=t_{del_{2}}\right)}\right]$ is computed as the error. Therefore, the profit is calculated for the divided $\frac{d_{d}}{d_{a}}$ in $\sum t_{t}$. The assignment of $\frac{d_{d}}{d_{a}}$ to the classified $t_{t}$ for ρ follows the order of priority to reduce the $t_{del}$ and thereby maximize $\frac{d_{d}}{d_{a}}$. The process of $\left(\frac{d_{d}}{d_{a}}\right)$ maximization based on the T for $\sum t_{t}$ is represented in Fig. 2(b), (c) and 2(d) respectively. In Fig. 2(a), the state transaction is illustrated.

In the above representation, the delay of the medical data delivery varies based on the state. The $t_{t}$ and e of the $\left(f\times d_{a}\right)$ is estimated on its concurrency and $t_{d}$. Therefore, for the state $\rho \left[h_{t_{t}+1}=t\right],t_{del}$ is less, if $\left(t_{t_{1}}-t_{t_{2}}\right)<t_{del_{1}}$ (as in Fig. 2 (b)). On the other hand, if the state is $\left[t_{t+1}=h_{t_{t+1}}\right]$ or $\left[t_{t+1}=t_{t}\right]$, then e≠0 and e=0 as the $\left(f\times d_{a}\right)$ is continuous and discrete if t→h. Hence, the T is retained in t and $t_{t+1}$ states for the varying $t_{t}$ and hence e is less. In this case, from Equation (3), $\rho \left[h_{t_{t}+1}=t\right]\left[t_{f_{t_{t}}}\right]$ as $\left[h_{f_{t_{t}}}\right]=0$ and $T\propto \rho \left[h_{t_{t}+1}=t\right]\cap \rho \left[t_{t_{t+1}}=t\right]$ identifying e if the state =2 [Fig. 2(b)]. The decision of state T relies on $\tau_{T}$ such the $d_{a}$ with max⁡{p} is mapped to the $T\propto \rho \left[t_{t_{t+1}}=t\right]$ state, followed by order of occurrence $\left[h_{t_{t+1}}=t\right]$. If such a scenario exists, the e observed is less [except state = 2], reducing the $t_{del}$. Fig. 2(e) presents the observed delay for the three states concerning the varying T.

Fig. 2(e) shows that the State = 3 achieves minor delay as the state is retained in the transmission. The other two cases require a switchover based on the availability of the dissemination slot. And therefore, the sequential dissemination of State = 3 requires less delay. In this state, no additional wait time is observed.

### 3.2 Data delivery modeling

As argued in the delay modeling process, the cases of state = 1 and state = 3 (with less e) can satisfy the objective in Equation (2). Therefore, these two cases need not be switched for their T states. Instead, the case of state = 2 (i.e.) $\rho \left[t_{t+1}=h_{t_{t}+1}\right]$ needs to be replaced with an appropriate state to satisfy Equation (1). For this purpose, the validation of $\tau_{T}$ is considered. The less in $d_{a}$ at the time $d_{d}$ is estimated for all f. In this case, $d_{d}$ is estimated using Equation (5).

(5) $$d_{d}=\prod_{i=1}^{f}\left(1-d_{a_{i}}\times e_{i}\right)$$

In Equation (5), the required $d_{d}$ is expected to improve the reliability of the dissemination techniques. It has been further derived by considering the consecutive medical records $d_{a_{i}}$ and the error value $e_{i}$ . The error value is computed as, $\left[\frac{t_{del_{1}}-t_{del_{2}}}{count\left(t_{del_{1}}=t_{del_{2}}\right)}\right]$. This computation helps to maximize the decisions $\prod_{i=1}^{f}\frac{\left(1-{d_{a}}_{i}\times e_{i}\right)}{\left(f\times d_{a}\right)}$. The decision-making process concerning state = 2 is balanced between $\left(f\times d_{a}\right)$ and e observed. If e is high, then $d_{d}$ is very less, reducing the quality of dissemination. Therefore, pre-estimation of the rate of $\left(d_{a}\times e\right)$ in all f is necessary to retain or change the state. The rate of data loss $\left(d_{l}\right)$ in e estimated as

(6) $$d_{l}=\left\{ \begin{array}{c} \left(\tau_{T}\times \frac{d_{a}}{f}\times t_{t}\right)-\left(d_{d}\times t_{d}\right),if\;e\neq 0\\ \frac{d_{a}\times t_{t}}{t_{del}}-\frac{d_{d}\times t_{d}}{t_{del}},if\;e=0 \end{array}\right.$$

**Fig. 3:**
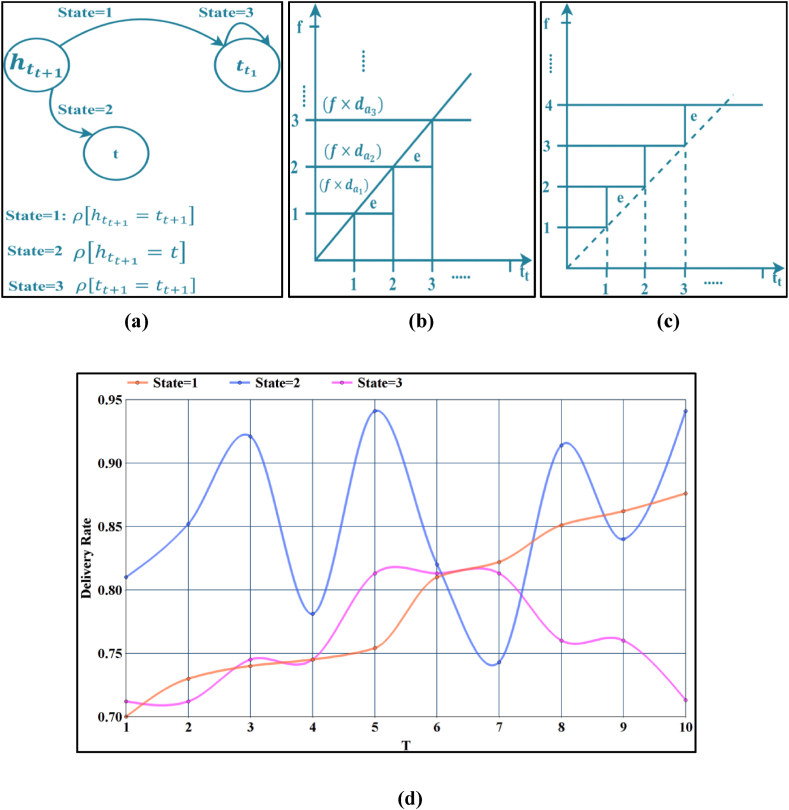
State diagram (a) State representation; (b) State −1 and 3 transactions and (c) State −3 transactions. (d): Delivery Rate for the varying T.

In Equation (6), the $d_{l}$ is defined as data loss which is estimated from the different parameters like pre-estimation rate, $d_{d},t_{d}$ . The two cases of $d_{l}$ in both e≠0 and e=0 requires to be addressed for improving the rate of $\frac{d_{d}}{d_{a}}$. The case of $d_{l}$ observed in e≠0 condition occurs due to state = 3 condition as $\left(f\times d_{a}\right)$ overlaps with the other data flows. Therefore, the delivery date to satisfy the condition in Equation (2) is verified for the state $\rho \left[t_{t+1}=h_{t_{t+1}}\right]$. In this state, if the rate of $d_{l}$ increases on a comparison concerning the $d_{l}$, dissemination channel/medium/infrastructure is replaced in the previously existing state. The change in dissemination medium increases the chance of $d_{d}$ under controlled $t_{del}$, if the $d_{l}$ in the early state is identified. The probability of change in state and its profit $\tau_{T}$ are to be balanced in determining the new state for the dissemination model. There are two probable chances for changing the state of $\rho \left[t_{t+1}=h_{t_{t+1}}\right]$ (i.e.) $h_{t_{t+1}}\to t$ or $h_{t_{t+1}}\to t_{t_{t+1}}$. In these two cases, if $h_{t_{t}+1}\to t_{t_{t+1}}$, then $\rho \left[t_{t+1}\right]$ is retained, and therefore the case of $h_{t_{t+1}}\to t$ is the second possible case. Hence, the state representative for $d_{l}$ Based dissemination is illustrated in Fig. 3 (a) by $d_{a}$. In Fig. 3(b) and (c), the allocation of $d_{a}$ based on the dissemination objective represented.

In Fig. 3(b) and (c), the output of the decision-making for f concerning $t_{t}$ over the varying $\left(d_{a}\right)$ is represented. In this state selection model, the $d_{d}$ is determined as

(7) $$d_{d}=\left\{ \begin{array}{c} \prod_{i=1}^{f}\left(1-d_{a_{i}}\times e_{i}\right)-\sum_{i=1}^{t+1}\frac{{\left(d_{a}\times t_{t}\right)}_{i}}{{t_{del}}_{i}},\forall e=0\;\&\&h_{t_{t+1}}=t\\ \prod_{i=1}^{f}\left(1-d_{a_{i}}\times e_{i}\right)-\sum_{i=1}^{t}\frac{{\left(d_{a}\times t_{t}\right)}_{i}}{t_{del_{i-1}}},\forall e=0\;\&\&h_{t_{t+1}}=t_{t+1} \end{array}\right.$$

In Equation (7) $d_{d}$ is estimated from the consecutive medical records $d_{a_{i}}$ and error value $e_{i}$. Here, the computations are taken for specific records t and next data t+1. In the above delivery estimation, the state = 3 follows the process discussed in the delay modelling process. Instead of selecting $t_{t+1}$ or t is the crucial process in this $d_{d}$ modelling. The factor of $e_{i}$ determines the state selection with the role of increasing or decreasing $d_{d}$ as represented in Fig. 3 (b) and 3 (c), respectively. Let $t_{i}$ and $t_{i+1}$ (increasing $d_{d}$), and $e_{i}\in \left[\frac{d_{d}}{t_{i}},\frac{d_{d}}{t_{i+1}}\right]$, the state is to be retained in $\rho \left[h_{t_{t+1}}=t\right]$ [contrary to Fig. 3(b)]. It is because the less $e_{i}$ cannot be guaranteed in the successive $t_{i}$ or $t_{i+1}$. Instead, if $e_{i}\in \left[0,\frac{d_{d}}{t_{i}}\right]\;or\;\left[0,\frac{d_{d}}{t_{i+1}}\right]$, the state of $\rho \left[h_{t_{t+1}}=t_{t+1}\right]$ is considered. Therefore, concerning the P of the wearable sensor data, the state T is assigned; for example, if the P is high, then $\rho \left[h_{t_{t+1}}=t_{t+1}\right]$ is assigned, else for a low-priority process of states and its variation based on $\tau_{T}$ and $e_{i}$ observed in each of the states. Therefore, the $e_{i}$ in the case of two $d_{d}$ [as in Equation (7)] is validated as

(8) $$e_{i}=\left\{ \begin{array}{c} {\left(1-\frac{t}{f}\right)}^{2}d_{d},if\;\rho \left[h_{t_{t+1}}=t\right]\\ \frac{d_{d}-d_{a}}{f},if\;\rho \left[h_{t_{t+1}}=t_{t+1}\right] \end{array}\right.$$

From the validation of $e_{i}$ as in Equation (8), the dissemination is modelled as, if $e_{i}\in \left[0,\frac{d_{d}}{t_{i}}\right],i\in f$, and then $\frac{d_{d}}{d_{a}}$ is high in $\left(t_{i}\;to\;t_{i+1}\right)$ interval. On the other hand, if $e_{i}\in \left[0,\frac{d_{d}}{t_{i+1}}\right]$, then dissemination is pursued through the state of $\rho \left[h_{t_{t+1}}=t_{t+1}\right]$ where $\left(t_{i}\;to\;t_{i+1}\right)$ generates high $d_{d}$ then the previous case. In Fig. 3(d), the delivery rate for the varying T is presented.

The change in T achieves a varying delivery rate, irrespective of its retained state. The delivery rate is improved if the transmission state is maintained. In this scenario, all three states are said to maintain transmission, wherein state = 2 (similar to the case of delay modelling) achieves a high delivery rate. There are fewer changeovers in this probability.

The joint modelling of delay and delivery-based WS data constraints are modelled for the CL components for f=1ton flows. These flows are independent of the available CL components before a unified transmission. The pre-modelling of delay and delivery for sensitive information/dissemination between WS and CL helps to improve the quality of handling sensor data concerning time and successful delivery.

## 4 Results and discussion

The performance of the proposed dissemination technique is assessed using a real-time human subject. The sensors for monitoring heartbeat, pulse, temperature, and blood pressure are considered in this assessment. Here the external configuration of the backbone network is similar to the one illustrated in Fig. 1. The CL consists of 4 infrastructure units unifying communication to a destination. The sensor placement is considered to be placed on the left and right side of a human body, and therefore, eight sensor devices are placed. The communication of the sensors is observed at different intervals transmitted in multiple flows to the CL. The maximum flows from the MD are 8/s. The time-to-live period of the sensor data is fixed as 64 ms, within which an acknowledgement to the MD is to be received. The MD can communicate with four access points connected to a laptop computer. The operating bandwidth of the MD is 2 Mbps, and it requires a wireless communication medium through Wi-Fi. The discussed system was developed using the.Net Framework with respective networking libraries and API. During this process, classes are created with respective healthcare scenarios to identify the respective classes and methods for CL, MD, and access point.

The performance of the proposed NDTDT is compared with the existing methods, such as DCSM [27], DDPT [30], and MEC-DT [34]. These methods are compared with the introduced NDTDT process because; the existing methods can perform effectively on specific applications. Due to the effective working procedures and results in this work, the methods are considered to compare the system. Here, these methods are utilized to process the sensor-based collected information, which gives particular results; however, these methods achieve minimum results compared to the NDTDT approaches. These methods are implemented the same way the NDTDT method is implemented, but the working procedures of each method are different. The working process is taken in the respective research authors' work. The impact of varying sensors and data flows over dissemination probability, delivery rate, and average delay is analyzed in the comparative analysis.

### 4.1 Impact of sensors

The density of the wearable sensor alters the data and its dissemination interval. The changes in the density of the sensors require different dissemination instances, with the changes in f. Therefore, $\left(f\times d_{a}\right)$ is not the same for all $t_{t}$. In the following study, results due to the different ranges of the physical wearable sensors presented the varying $\left(f\times d_{a}\right)$. The optimality of the proposed dissemination technique is assessed using the above metrics. In varying factor 1 based on the impact of sensors, three metrics are evaluated i) Dissemination Probability and Sensors-considering the need for flawless transmission and the density of the sensors. It entails arranging transmission slots with various states and limits according to the profit and importance of sensor data. ii)Delivery Rate and Sensors-It addresses variations in sensor density by allocating numerous dissemination time instances and tailoring delivery based on goals. For delay modeling, the T states and limitations are considered, and certain circumstances are chosen for data transmission. The research considers various instances for e scenarios to determine possible delivery rates. iii)Average Delay and Sensors-The suggested method finds and filters workable time instances to delay limitations. For each slot designated for dissemination, the delay is taken into account. The classification and prioritization of dissemination states based on profit help to minimize delay in the suggested strategy.

#### 4.1.1 Dissemination Probability and Sensors

**Fig. 4:**
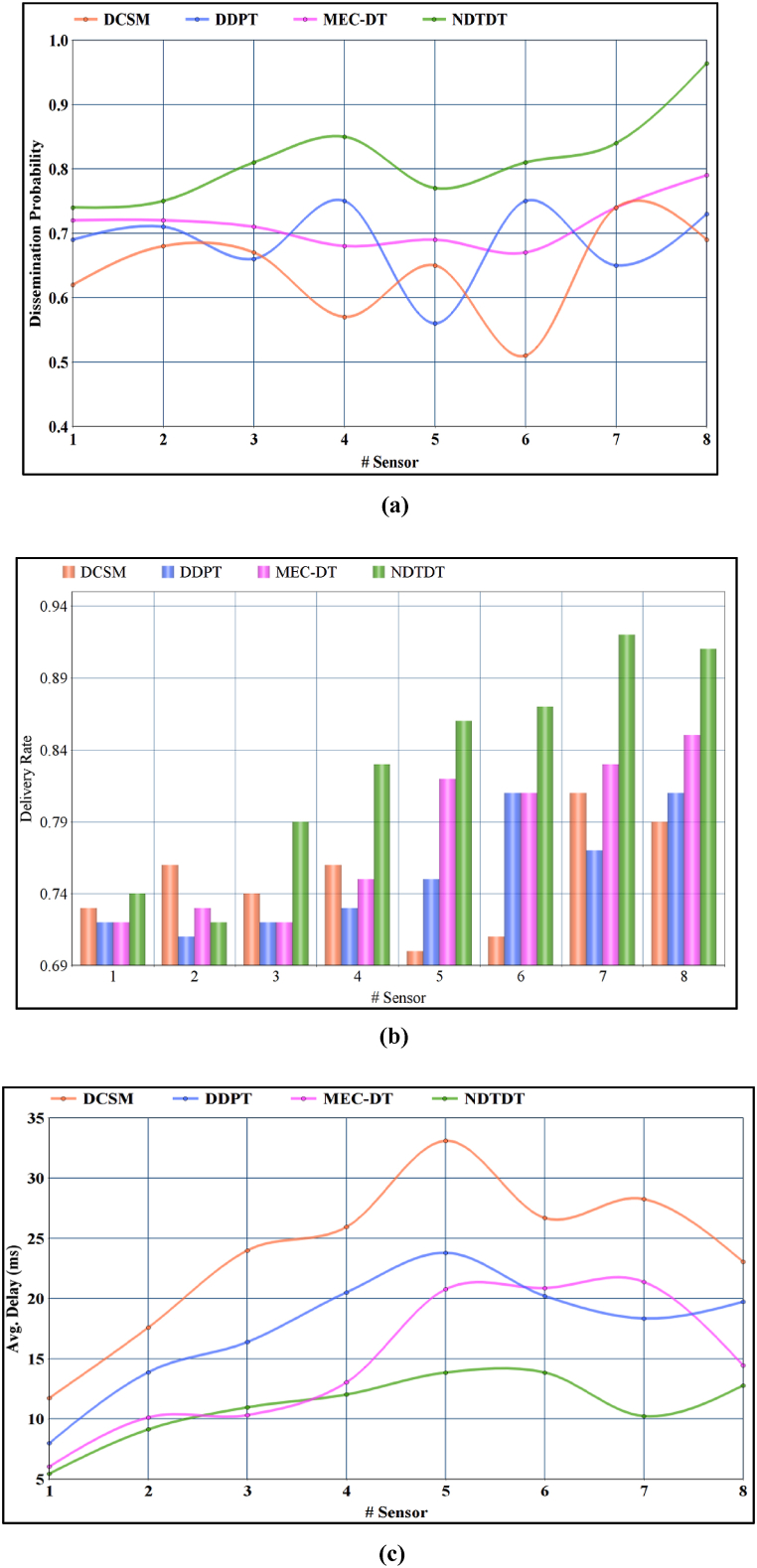
**(a):** Dissemination probability. **(b):** Delivery rate. **(c):** Avg. Delay.

The need for dissemination increases over different time Intervals as the density of the sensor increases. The delay and data delivery states are independently modelled in the proposed technique. Allocating seamless transmission slots by satisfying constraints, as in Equation (2), is based on the profit and priority of the sensor data $t_{del}$. For any number of sensors, state = 1, 3 generates available slots for disseminating multiple f from the n sensors. The flow increases of the dissemination level exceed the available sensors. The T defined for ″h″ and sensor data relies on the relationship of $T\propto \rho \left[h_{t_{t+1}}=t\right]\cap \rho \left[t_{t+1}=t\right]$ such that e=0 for the state = 1 and 3 T. Instead, the e in the state = 2 (i.e.) $\rho \left[t_{t+1}=h_{t_{t+1}}\right]$ is balanced by identity max{P} and mapping it to the dissemination state of $\rho \left[t_{t_{t+1}}=t\right]$. It increases the chances of dissemination from $2t_{t+1}$ to $2t_{t+1}+\left(t+t_{t+1}\right)=3t_{t+1}+t$ is the possible dissemination performed. Hence, the dissemination probability of the proposed technique is high [Refer to Fig. 4 (a)].

#### 4.1.2 Delivery Rate and Sensors

The dissemination probability of NDTDT is high, demanding maximum delivery of medical data packets for reliable communication. The varying sensor density is satisfied by assigning multiple dissemination time instances, in which objective-based delivery modelling maximizes this matric $\frac{d_{d}}{d_{a}}$. The change in T states is considered for the non-feasible case of delay modeling from which the T sustaining conditions are selected for data delivery. Considering the constraints in Equation (2) and the precise validation of $e_{i}$ [using Equation (8)] helps to identify the $d_{d}$ for both e≠0 and e=0, wherein e=0 analyzed under $h_{t_{t+1}}=t$ and $t_{t+1}=t_{t+1}$ state transactions. As the $e_{i}$ is reduced from $\left[\frac{d_{d}}{t_{i}},\frac{d_{d}}{t_{i}+1}\right]$ to $\left[0,\frac{d_{d}}{t_{i}}\right]$ or $\left[0,\frac{d_{d}}{t_{i+1}}\right]$, $\rho \left[h_{t_{t+1}}=t_{t+1}\right]$ state, the delivery in this T is highly achievable [Refer Fig. 4 (b)].

#### 4.1.3 Average Delay and Sensors

Fig. 4(c) shows that the varying sensors' average delay is presented as a comparison. The constraints of $t_{del_{1}}=t_{del_{2}}$ or $t_{del_{1}}-t_{del_{2}}<t_{t}$ is satisfied by identifying all possible T and filtering $\left[h_{t_{t+1}}=t\right]$ for normalized dissemination, retains the delay. Delay is included for all the allocated dissemination slots of the varying sensors. The time-based profit of $\tau_{T}$ helps to map max{p} to the more prominent T state, in a view to satisfying the delay objective. Classifying state = 2 and three based on $\left(f\times d_{a}\right)$ from the n sensors and consolidating the dissemination $\rho \left[h_{t_{t+1}}=t\right]=\left[t_{t+1}\right]$ and $T\propto \rho \left[h_{t_{t+1}}=t\right]\cap \rho \left[t_{t+1}=t\right]$ aids delay minimization in the proposed technique.

### 4.2 Impact of data flows

**fig. 5:**
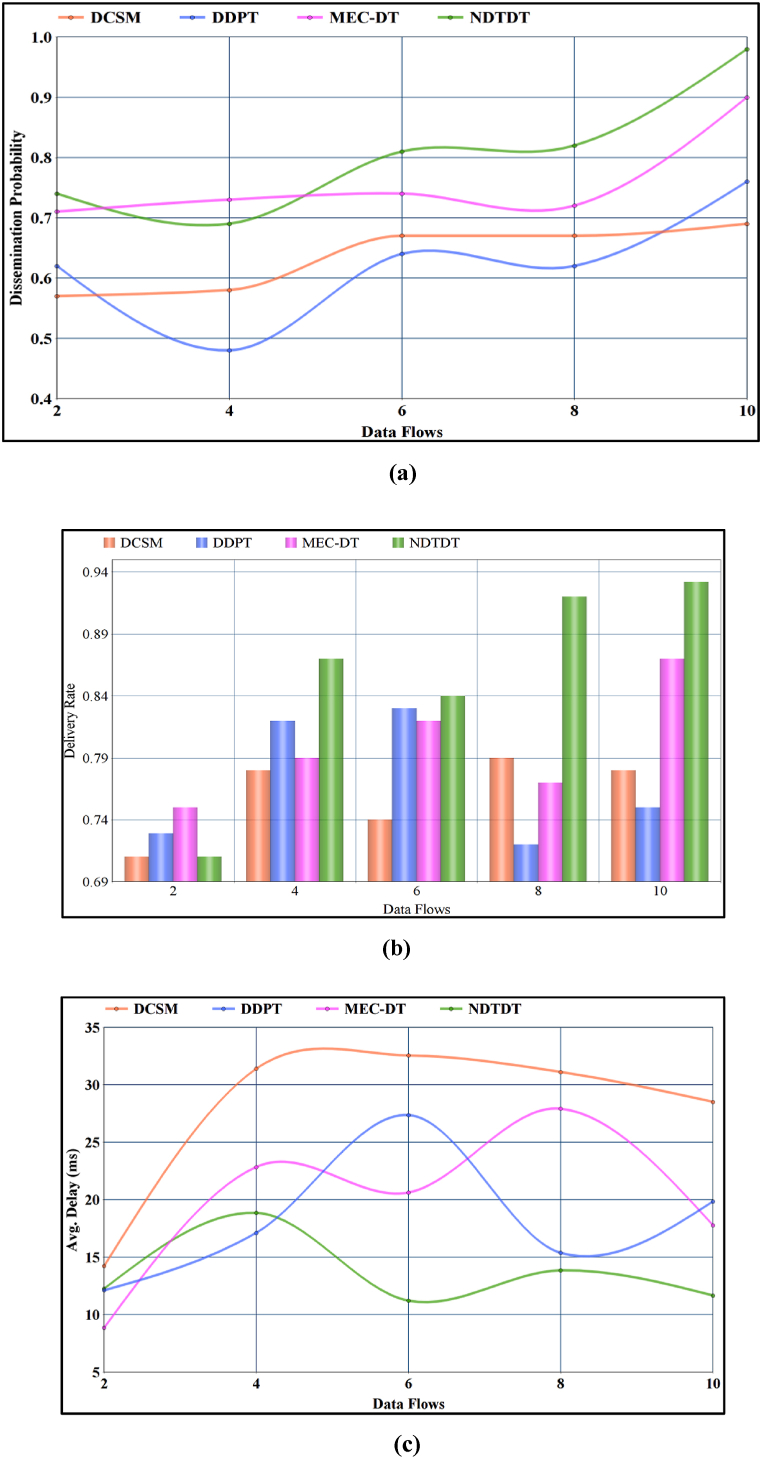
**(a):** Dissemination probability. **(b):**Delivery rate. **(c):** Avg. Delay.

The varying sensors increase the rate of data flow if the available dissemination instances are high $\left(f\times d_{a}\right)$. For the varying data flows, the size of the medical data needs to consider along with P and $e_{i}$ in both delay and delivery modelling. The impact of these factors helps to manage the delivery and dissemination of the $d_{a}$. The varying f needs to be verified for all the incoming $d_{a}$ to improve the delivery rate and reduce delays. fig. 5 (a), 5 (b), and 5 (c) represents the impact of data flows over dissemination probability, delivery rate, and delay. For varying data flows, the number of sensors used initially is 8, and it varies based on the aggregation priority where each sensor is fixed with 64 ms time to live period before it terminates; the communication to the mobile device has to happen. Based on its varying density with high dissemination instances over a time interval, it affects the delivery of data flow. The data flow rate is considered with time delay and delivery modeling since the number of sensor counts has varied in the execution time based on its working and lifetime value.

#### 4.2.1 Dissemination probability and data flows

Data flow management in the proposed technique based on the availability state is the key factor for improving the dissemination probability. The factors $d_{d}$ and $d_{l}$ are estimated in forehand that is further approximated by identifying $e_{i}$ and its range. The approximation of $e_{i}\left[\frac{d_{d}}{t_{i}},\frac{d_{d}}{t_{i}+1}\right]$ from $\left[0,\frac{d_{d}}{t_{i}}\right]$ or $\left[0,\frac{d_{d}}{t_{i+1}}\right]$ helps to increase the number of flows irrespective of the transmission time instances. Therefore, the availability of transmission instances are high in the proposed technique. Besides, the refined state of $\rho \left[t_{t+1}=h_{t_{t+1}}\right]$ (i.e.) $\rho \left[h_{t+1}=t\right]$ and retained $\rho \left[h_{t+1}=t_{t+1}\right]$ helps to allocate instant dissemination time intervals for $d_{a}$. Hence, the probability of the occurring/allocated dissemination is high in the proposed technique. For wearable sensor data to be performed more effectively by overlapping transmissions and improper slots distribution, the dissemination limitations are addressed from a delay and delivery perspective. The performance analysis demonstrates the reliability of the technology proposed by improving the delivery rate and the probability of diffusion by reducing uncertainty.

#### 4.2.2 Delivery rate and data flows

If the aggregated $d_{a}$ is successfully disseminated, then $d_{d}$ is high, augmenting the need for allocating multiple dissemination instances. On the other hand, if the dissemination is continuous, then e increases [40]. To mitigate the interference of e, the allocated dissemination instances are maximized for its $d_{d}$ in any t or (t+1). Based on the estimated $d_{d},d_{l}$ and e, the available state (state as in delivery modelling) is refined. Leaving out $\rho \left[h_{t_{t+1}}=t_{t+1}\right]$ and $\rho \left[t_{t+1}=t_{t+1}\right]$, the halting state of $\rho \left[t_{t+1}=h_{t_{t+1}}\right]$ is assigned. $\rho \left[h_{t_{t+1}}=t\right]$ to prevent unnecessary dissemination losses. The number of availability dissemination instances are validated if the halting state is observed. If this state is observed, then based on $d_{d}$ approximation [as per Equation (7)], the state of t or $t_{t+1}$ is retained. Therefore, in both the allocated t and t+1 states from $h_{t+1}$ increases the delivery rate.

#### 4.2.3 Average delay and data flows

The allocated Data Flows are moderate in both time and delivery-dependent modelling. The number of flows that are determined by $d_{a}$ is modelled with both delay and delivery metrics. In these cases, the non-optimal states (i.e.) $\rho \left[t_{t+1}=h_{t_{t+1}}\right]$ (as in delay modelling) and $\rho \left[h_{t_{t+1}}=t\right]$ are validated for improving the feasibility of $d_{a}$ dissemination. In the first case, if $\rho \left[h_{t_{t+1}}=t\right]$ is retained by discarding $\left[h_{t_{t+1}}\right]=0$ by exploiting the condition of $T\propto \rho \left[h_{t_{t+1}}=t\right]\cap \rho \left[t_{t+1}=t\right]$ for the delayed $d_{a}$. Similarly, in the delivery modelling process, the period estimation of $e_{i}$ and its range helps to retain the approximation of $d_{d}$ [as in Equations (7), (8)], reducing the need for re-dissemination due to dropouts [40]. Therefore, the two flexible conditions and validation factors help to retain the delay of the proposed dissemination technique. In Table 2, the performance of the comparative study is presented.

**Table 2:** Performance comparison.

Varying Factor	Metric	DCSM	DDPT	MEC-DT	NDTDT
Sensors	Dissemination Probability	0.69	0.73	0.79	0.964
	Delivery Rate	0.79	0.81	0.85	0.91
	Avg. delay (ms)	23.06	19.73	14.45	12.78
Flows	Dissemination Probability	0.69	0.76	0.9	0.98
	Delivery Rate	0.78	0.75	0.87	0.932
	Avg. delay (ms)	28.52	19.84	17.78	11.67

From Table 2 observation, the performance of the proposed NDTDT is seen to be reliable by achieving less delay for a better delivery rate.

## 5 Conclusion

The Non-Delay Tolerant Dissemination Technique (NDTDT) is suggested in the research as a useful technique for handling communication dependability in wearable sensor-enabled healthcare applications. Important problems with slot synchronization and congestion flow in data transmission are addressed by the NDTDT method. The model improves the overall efficiency of wearable sensor systems in monitoring patient health by minimizing dissemination delays and maximizing successful message transmission. Several important goals are outlined in the paper, including preventing overloaded dissemination, facilitating the delivery of urgent messages, decreasing delay and transmission losses, maximizing the delivery of aggregated wearable sensor information, and addressing slot synchronization and congestion flow issues. The method chooses suitable slots for handling WS information to reduce interruptions and transmission losses.

Additionally, it emphasizes maximizing the efficient delivery of gathered WS data and decreasing dissemination delays. The performance of the proposed NDTDT system is validated with two variants, sensors and data flows. The model improves the dissemination probability by 0.964% and 0.98, the delivery rate by 0.91 and 0.932, and the final metrics involved are an average delay of 12.78 ms and 11.67 ms. Heart rate sensors (>100 bpm) with high accuracy are best suitable for stress management but require high-precision measurements for cardiovascular tracking. Temperature sensor with high accuracy is useful for fever monitoring (>38 °C); if it lacks accuracy not applicable for critical healthcare. For the management of hypertension(low as <80 mmHg and high as >120 mmHg) and cardiovascular disorders(normal 95% or higher O_2_ consumption), the high accuracy and precise reliability of blood pressure sensors are essential for implementation.

The future scope may involve the hybrid NDTDT technique shows potential for extending the period for information transmission while reducing computational challenges. The hybrid method can further optimize the transmission of wearable sensor data in healthcare applications by using cutting-edge compression algorithms, edge computing capabilities, and leveraging upcoming technologies.

## Author contribution statement

Mohd Anjum: Sana Shahab: Conceived and designed the experiments; Performed the experiments; Analyzed and interpreted the data; Contributed reagents, materials, analysis tools or data; Wrote the paper. Shabir Ahmad: Conceived and designed the experiments; Analyzed and interpreted the data; Contributed reagents, materials, analysis tools or data; Wrote the paper and proofread the paper. Taegkeun Whangbo: Conceived and designed the experiments; Contributed reagents, materials, analysis tools or data and supervised this work.

## Data availability statement

Proposed methodology is assessed over the real time data.

## Additional information

No additional information is available for this paper.

## Funding

This work was supported in parts by the 10.13039/501100013173GRRC program of Gyeonggi province. [GRRCGachon2023(B02), Development of Medical Service Technology and by the National Research Foundation of Korea (NRF) Grant funded by the Ministry of Education under Grant NRF-2021R1I1A1A01045177.

## Declaration of competing interest

The authors declare that they have no known competing financial interests or personal relationships that could have appeared to influence the work reported in this paper.
